# Single-site laparoscopic high ligation of the extraperitoneal hernia sac with an epidural needle for incarcerated ovarian hernia in infants

**DOI:** 10.1186/s12893-022-01520-3

**Published:** 2022-02-23

**Authors:** Yun-jin Wang, Liu Chen, Qi-liang Zhang, Jian-qin Zhang, Xu Cui, Chao-ming Zhou

**Affiliations:** Department of Pediatric Surgery, Fujian Provincial Maternity and Children’s Hospital of Fujian Medical University, Fuzhou, 350001 People’s Republic of China

**Keywords:** Incarcerated ovarian hernia in infants, Single-site laparoscopy, Epidural needle, High ligation of hernia sac

## Abstract

**Background:**

The purpose of this study was to evaluate the safety and efficacy of single-site laparoscopic extraperitoneal hernia sac ligation with an epidural needle for incarcerated ovarian hernias in infants and young children.

**Methods:**

The clinical data of 38 infants with incarcerated ovarian hernias who underwent single-site laparoscopic extradural needle extraperitoneal hernia sac ligation from January 2015 to January 2018 were retrospectively analysed.

**Results:**

All procedures were successfully performed using laparoscopy with no need for conversion to open surgery. The time of hospital stay was 1.30 ± 0.39 days. During hospitalization and follow-up, there were no complications, such as intestinal or bladder injury, abdominal wall vascular injury, ovarian atrophy, hernia recurrence or contralateral indirect hernia. However, three patients experienced complications, including two cases of poor healing of the umbilical incision and one case of suture granuloma.

**Conclusions:**

Single-site laparoscopic high ligation of the extraperitoneal hernia sac with an epidural needle is a safe and feasible method for the treatment of incarcerated ovarian hernias in infants and young children. It has the advantages of minimal trauma, no scarring and good cosmetic effects.

## Background

An incarcerated inguinal hernia is formed by abdominal organs that cannot be returned after entering the hernia sac and remain in the hernia sac. It is the most common complication of inguinal hernia in children. If it is not handled in time, it can lead to ischaemia and necrosis of the hernia contents, resulting in serious consequences. The characteristics of embryonic development and inguinal anatomy in female children are different from those in male children. A small evagination of the parietal peritoneum, called the canal of Nuck, accompanies the round ligament through the inguinal canal to the labium majorum [1]. The canal of Nuck obliterates shortly before birth; however, its persistence allows herniation of the visceral contents into the canal [2]. In addition to the intestinal tube and omentum, most of its contents are the ovaries, fallopian tubes and even the uterus. Due to the small pelvic volume and the inclination of uterine accessories in female children, it is easy to approach the orifice, and therefore, the incidence of ovarian hernia is high. After strangulation, serious complications are often caused by ischaemia of the hernia contents. Traditional incarcerated ovarian hernia surgery requires dissection of the inguinal canal, causes some damage the normal anatomical structure of the inguinal area, with the disadvantages of greater surgical injury and more obvious scarring. With the continuous development and innovation of minimally invasive technology, laparoscopic surgery for incarcerated ovarian hernias has gradually been applied in clinical practice [3–6]. Here, we retrospectively analysed the clinical data of 38 infants with ovarian incarcerated hernias who underwent single-site laparoscopic high ligation of the extraperitoneal hernia sac with an epidural needle and evaluated the safety and effectiveness of this procedure in the treatment of ovarian incarcerated hernias in infants and young children.

## Methods

This study was approved by the ethics committee of Fujian Maternity and Child Health Hospital, Affiliated Hospital of Fujian Medical University, and strictly adhered to the tenets of the Declaration of Helsinki. All patients’ guardians signed an informed consent form before the operation.

### Patients

Patients met the inclusion criteria if they presented with incarcerated ovarian hernia. Patients were excluded from this study for the following reasons: (1) their general condition was poor, with a history of peritonitis and systemic poisoning symptoms or serious medical diseases such as cardiopulmonary dysfunction; (2) they had a history of abdominal surgery; (3) they had a recurrent inguinal hernia; (4) their incarcerated ovaries were necrotic and needed to be replaced by ovariectomy; or (5) they refused to consent to the operation or cooperate during the follow-up schedule.

A total of 46 patients with incarcerated ovarian hernia in our hospital from January 2015 to January 2018 were included in this study. Among them, 8 patients were excluded from the study for the following reasons: 4 with a poor general condition, 1 with a history of abdominal surgery, 1 with a recurrent inguinal hernia and 2 with necrotic ovaries. Finally, this study retrospectively analysed the clinical data of 38 patients, including preoperative general data and intraoperative, postoperative and follow-up data. All patients were diagnosed by physical examination, combined with clinical manifestations and inguinal colour Doppler ultrasonography, and manual reduction failed in all cases. There were a total of 13 patients with left hernia and 25 patients with right hernia, and the average age and weight of the patients were 5 months (21 days to 11 months) and 5.6 kg (2.7–11.1 kg), respectively (Table 1). A routine clinical examination was performed before the operation, including an electrocardiogram, chest radiography and blood examination. All patients received preoperative bowel preparation with enemas. All patients underwent single-site laparoscopic high ligation of the extraperitoneal hernia sac with an epidural needle.

**Table 1:** Clinical data of the patients in this study

Item	
Number of patients	38
Age, median (range)	5 months (21 days–11 months)
Weight, median (range)	5.6 kg (2.7–11.1 kg)
No. transcrotalorchidopexies	
Left side	13 (34.2%)
Right side	25 (65.8%)
Operation time, median (range)	
Unilateral	32 (26–41) min
Bilateral	45(40–56) min
Time of hospital stay	(1.30 ± 0.39) days
Duration of follow-up, median (range)	1 years (3 months–3 years)
Incision length, median (range)	1.1 (0.8–1.5) cm

### Procedure

**Fig. 1:**
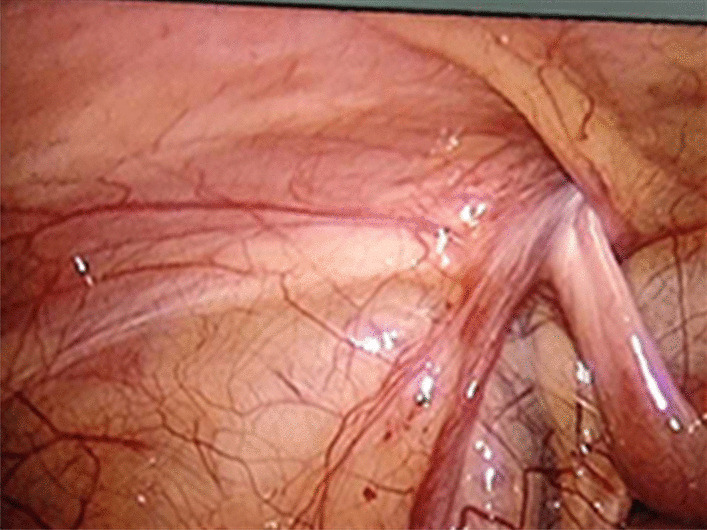
The oviduct continued to the inguinal canal with slight oedema and hyperaemia

**Fig. 2:**
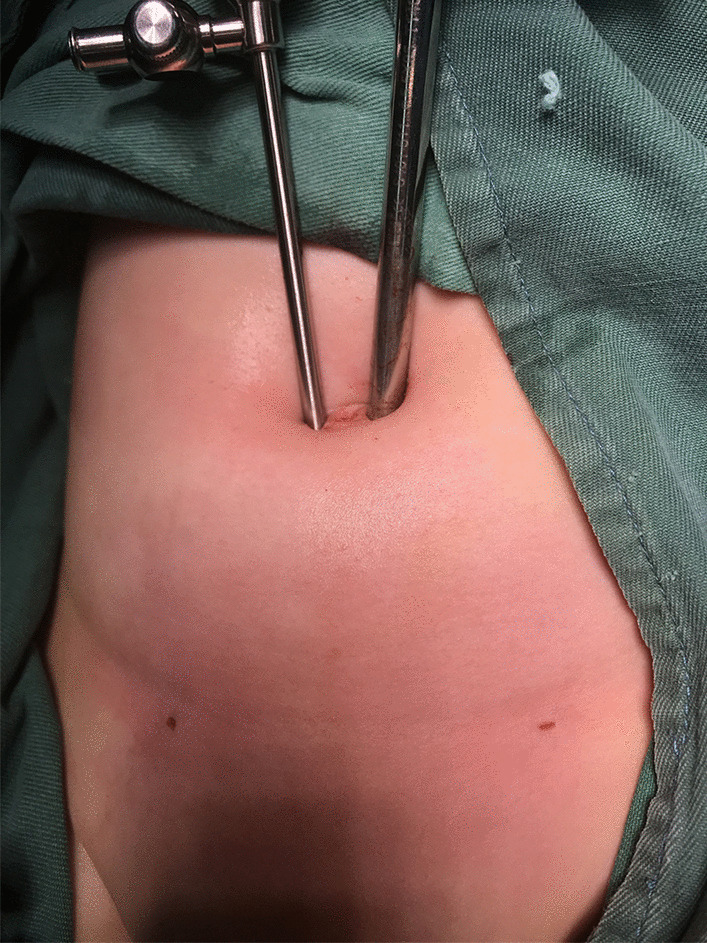
A 3-mm trocar was placed around the umbilical ring and grasping forceps were inserted

**Fig. 3:**
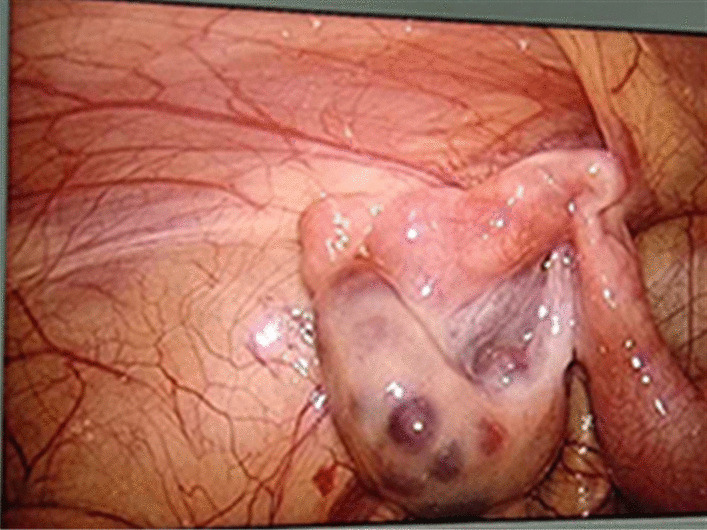
The colour of the ovaries was slightly purple, with good blood supply and no necrosis

**Fig. 4:**
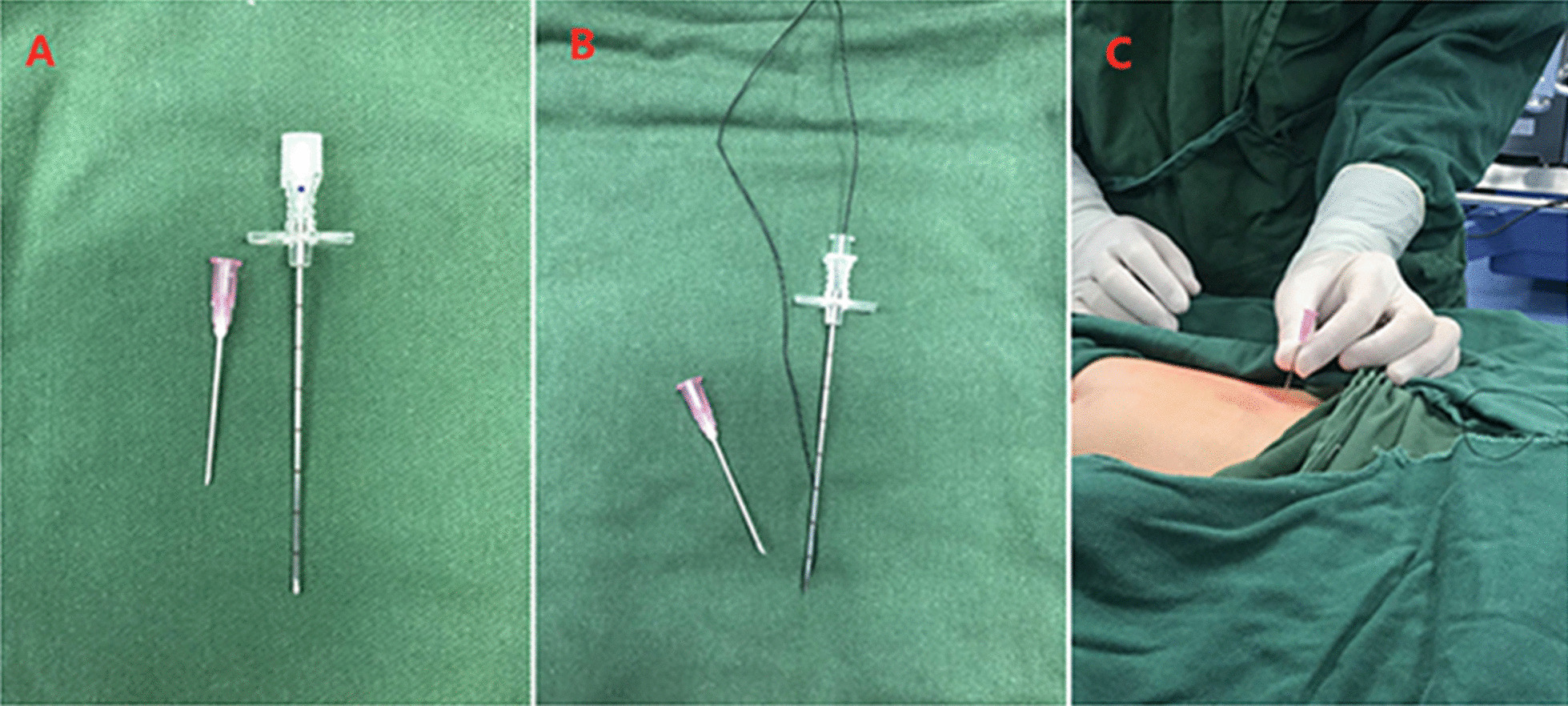
**A** The needle of a No. 12 syringe. **B** Epidural puncture needle with double-stranded 2-0 nonabsorbable braided sutures. **C** The skin was punctured at the unclosed side of the skin by the needle of a No. 12 syringe

**Fig. 5:**
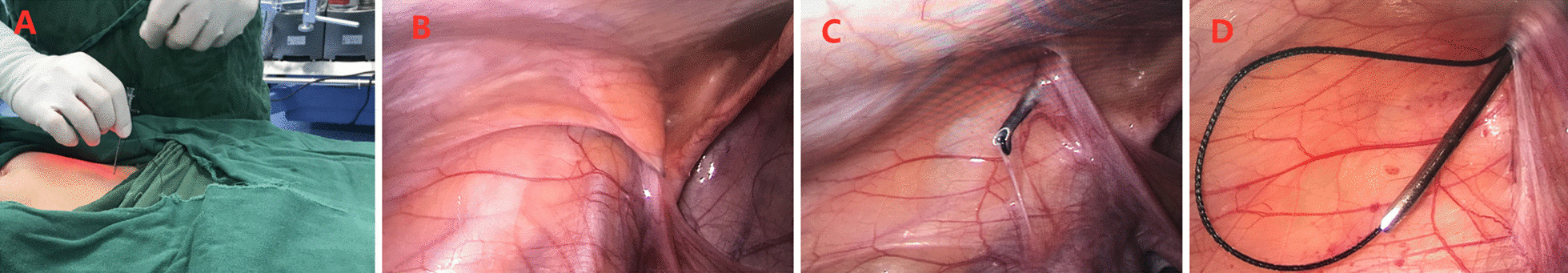
**A**, **B** The epidural puncture needle was inserted into the anterior wall of the inguinal canal through the anchor point. **C** The needle was advanced extraperitoneally on the medial side of the ring. **D** The needle was gently withdrawn until its tip reached the roof of the internal ring, with the loop remaining in the cavity

**Fig. 6:**
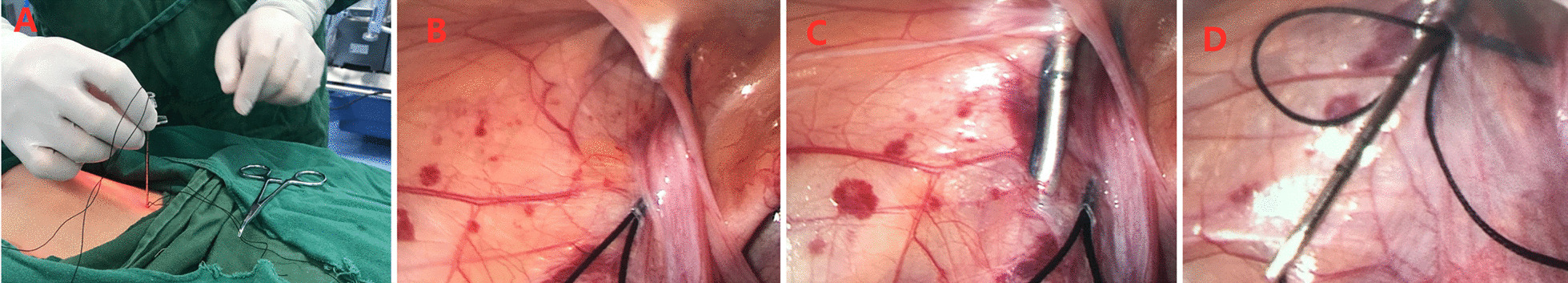
**A** The epidural puncture needle was pierced into the front wall of the inguinal canal again through the anchor point. **B**, **C** The epidural needle was advanced along the lateral side of the ring and passed into the suture loop in the cavity at the same peritoneal puncture site. **D** A long suture loop was sent into the peritoneal cavity through the sheath of the epidural needle in an antegrade manner

**Fig. 7:**
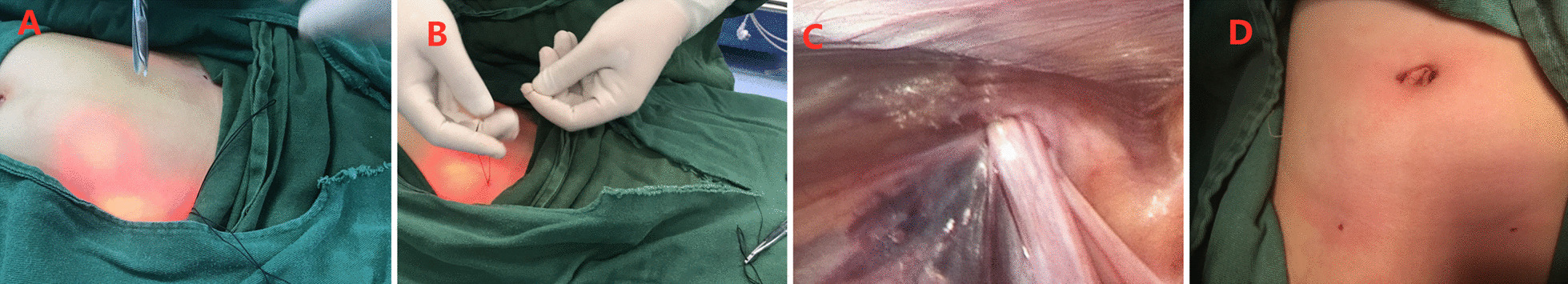
**A** The long suture loop was pulled out through the abdominal wall by picking up the first suture loop. **B**, **C** The internal ring was closed by knotting the suture extracorporeally. **D** Incision postoperation

All procedures were performed under general anaesthesia with orotracheal intubation. An approximately 5 mm layer-by-layer incision in the umbilical skin was made, and a 5 mm trocar was placed directly into the abdomen to establish a pneumoperitoneum (8–10 mmHg). We then explored the abdominal cavity: a small amount of clear ascites was found in the abdominal cavity, the ring of the affected inguinal canal was not closed, and the oviduct continued to the inguinal canal with slight oedema and hyperaemia (Fig. 1). Under the condition of laparoscopic enlargement, anatomical structures such as the bilateral internal ring orifice and inferior epigastric artery can be clearly identified. A 3-mm trocar was placed around the umbilical ring, and grasping forceps were inserted (Fig. 2). The tension of the fallopian tube was maintained by lifting the fallopian tube lightly. Manipulation was performed in vitro (soft skin mass at the groin) to return the incarcerated ovaries. The colour of the ovaries was slightly purple, with good blood supply and no necrosis (Fig. 3). Under the guidance of a laparoscope, the skin was punctured at the unclosed side of the skin by the needle of a No. 12 syringe (Fig. 4), and the epidural puncture needle with a double-stranded 2-0 non-absorbable braided suture was inserted into the anterior wall of the inguinal canal through the anchor point (Fig. 5A, B). The needle was advanced extraperitoneally on the medial side of the ring (Fig. 5C). Subsequently, the epidural needle was gently withdrawn until its tip reached the roof of the internal ring, with the loop remaining in the cavity (Fig. 5D). The epidural puncture needle was pierced into the front wall of the inguinal canal again through the anchor point with a double-stranded 2-0 nonabsorbable braided suture (Fig. 6A). The needle was advanced along the lateral side of the ring and passed into the suture loop in the cavity at the same peritoneal puncture site (Fig. 6B, C). Aided by the epidural needle and its core, a long suture loop was sent into the peritoneal cavity through the sheath of the epidural needle in an antegrade manner (Fig. 6D). The laparoscope was then inserted into the long suture loop so that the loop could be fixed in the cavity when the needles were withdrawn from the abdominal wall. Finally, the long suture loop was pulled out through the abdominal wall by picking up the first suture loop (Fig. 7A), and the internal ring was closed by knotting the suture extracorporeally (Fig. 7B, C). If there was an occult hernia on the opposite side, it was treated together. After closing the inner ring, we carefully checked the abdominal cavity for bleeding, released the peritoneal gas, and removed the trocar before suturing the umbilical incision (Fig. 7D).

## Results

Thirty-eight cases of ovarian hernia (including 13 cases on the left side and 25 cases on the right side, as well as 24 cases with an unclosed contralateral inguinal ring) successfully underwent laparoscopic high ligation of the extraperitoneal hernia sac with an epidural needle under single-site laparoscopy. In this group, seven cases were incarcerated for a long period of time, and the suspected disorder of incarcerated ovarian blood supply was found during the operation. After laparoscopic-assisted reduction, the observation time was prolonged, and the blood supply to the ovaries recovered. The operative times of the unilateral and bilateral ovarian hernias were 32 (26–41) min and 45 (40–56) min, respectively. The time of hospital stay was 1.30 ± 0.39 days (Table 1). During hospitalization and follow-up, there were no complications, such as intestinal or bladder injury, abdominal wall vascular injury, ovarian atrophy, hernia recurrence or contralateral indirect hernia. However, three patients experienced complications, including two cases of poor healing of the umbilical incision and one case of suture granuloma. All patients were followed up for 1 year. The follow-up time points were 1 week, 1 month, 3 months, 6 months and 1 year after the operation. Outpatient follow-up was used to follow up on the symptoms and signs of the children. All patients underwent abdominal ultrasonography 6 months and 1 year postoperatively to check the ovarian status, and the prolapsed ovary appeared morphologically normal and with a good vascular supply in all operated patients.

## Discussion

Incarcerated ovarian hernia is a common type of incarcerated inguinal hernia and one of the emergency diseases in paediatric surgery. As recently reported [7, 8], the incidence of incarcerated ovarian hernia ranges between 6 and 15% of all inguinal hernias in female infants.

For female infants with incarcerated ovarian hernias, emergency surgical treatment is needed when manual reduction fails. The traditional surgical approach is to perform high ligation of the hernia sac through the inguinal incision. For children with incarcerated hernias, local inguinal tissue congestion and oedema are obvious, and the operation is difficult. To reduce the difficulty of the operation and tissue identification, the surgical incision is often larger than that of non-incarcerated hernia, the structure of the inguinal canal is more traumatic, and the scar is more obvious after operation. Family members of female children often have higher requirements for postoperative cosmetology. Meanwhile, traditional operations cannot detect and properly address occult contralateral inguinal hernias, while bilateral and occult contralateral inguinal hernias are more common in infants and young children’s inguinal hernias [9, 10]. Zhou reported that the incidence of contralateral inguinal hernias was 21% [11]. Zampieri reported that contralateral exploration should be routinely performed in girls with inguinal hernia until they reach 4 years of age [12]. Gollu reported that transinguinal laparoscopic exploration of the contralateral inguinal hernia clearly and significantly reduced the need for surgery for a metachronous hernia at a later date [13]. Some of these patients had symptoms that appeared late and thus often required secondary surgery. When inguinal hernia repair is performed through a laparoscopic approach, these occult hernias may be easily addressed during the same operation without additional skin incisions. This may ultimately prevent the morbidity of developing a metachronous hernia that requires repair [14]. This process can avoid the pain of a secondary operation and reduce the economic burden [15]. The advent of minimal access techniques has revolutionized the traditional management of inguinal hernias [16]. With the development of endoscopic equipment and technology, the laparoscopic approach is gaining popularity because of the potential advantages of faster recovery, attenuated pain, improved cosmesis, and low recurrence rate [17–19].

Laparoscopic high ligation of the extraperitoneal hernia sac with an epidural needle is a simple, reliable and simple puncture technique that can be accomplished by external ligation. It has the advantages of minimal trauma, fast recovery, low recurrence rate and good cosmetic effects [20–24]. Compared with traditional high ligation of the hernia sac, it has the following advantages. First, female infants with an ovarian incarcerated hernia have obvious local tissue congestion and oedema, and traditional surgery is prone to damaging local tissues, such as the ovaries and fallopian tubes. Laparoscopy does not dissect the structure of the inguinal canal, avoiding the influence of local tissue congestion and oedema on the operation. Second, Shalaby et al. [25] reported that incarcerated hernias are easier to reposition under laparoscopy because the hernia contents are pulled by grasping forceps without damage to the abdominal cavity, and then the hernia contents are repositioned by an external technique. In addition, the pneumoperitoneum pressure enables carbon dioxide to be blown into the inner ring mouth, thereby expanding the inner ring mouth. Among the 38 children in this group, the incarcerated hernias were not reduced before surgery. The application of laparoscopy with an operation channel for non-invasive grasping forceps can enlarge the internal ring mouth and assist in pulling the hernia contents. The incarcerated hernia is relatively easy to reduce. Third, an occult contralateral inguinal hernia can be found under laparoscopy and treated simultaneously [5, 26]. In this group, occult contralateral inguinal hernias were found in 24 children during the operation, and these children underwent high ligation of the hernia sac to prevent the risk of reoperation caused by the contralateral hernia. Fourth, the ovary is an important endocrine organ in women and is also the source of female reproductive cells, which play a very important role in women’s lives. Therefore, when an incarcerated inguinal hernia occurs in a female child and the hernia contents are suspected to be the ovaries, it is extremely important to judge the nature and vitality of the hernia contents in a timely manner to guide subsequent treatment. Laparoscopy can accurately observe the reduction process of the incarcerated hernia contents [27]. It is clear that there is no damage to the ovary after reduction. If ovarian ischaemia and necrosis are found, urgent treatment should be provided to prevent the serious consequences of blind manual reduction in children [3]. Fifth, under laparoscopy, the hernia sac is ligated at a higher position through external operation of the epidural puncture needle, and no space is left for the suture of the inner ring orifice, which is helpful to prevent recurrence after operation [28]. Sixth, the incision is concealed, there is no obvious scarring after the operation, and the cosmetic effect is good, which can reduce feelings of inferiority that may occur in the psychological development of female children and is more readily acceptable by their families. Seventh, because laparoscopic operation can treat the inner ring orifice simply under direct vision, it reduces the difficulty of operation and shortens the postoperative course and operative time. Recovery after laparoscopic surgery is fast, which reduces the timing of discharge.

Although this retrospective study had a certain size, there are still several limitations. First, this was a retrospective study with a limited number of patients from a single centre, and more research from multiple centres is needed to assess the effectiveness and complications of this technique. Second, the median follow-up duration was relatively short, and a longer follow-up period is warranted.

## Conclusion

In conclusion, single-site laparoscopic high ligation of the extraperitoneal hernia sac with an epidural needle for ovarian incarcerated hernias in infants is a safe and feasible alternative to conventional surgery. The cosmetic results were impressive, leading to less physical and psychological trauma, and the follow-up results were promising.

## Data Availability

The datasets of the current study are available from the corresponding author upon reasonable request.
